# Sampling and analysis of airborne ammonia in workplaces of China

**DOI:** 10.1002/1348-9585.12100

**Published:** 2019-11-25

**Authors:** Zhizhen Xu, Ling Guo, Dongxu Wang, Zhe Bi, Zhaohui Fu

**Affiliations:** ^1^ Beijing Municipal Institute of Labor Protection Beijing PR China; ^2^ National Institute of metrology, China Beijing PR China

**Keywords:** airborne, ammonia, determination method, workplaces

## Abstract

**Objectives:**

With the increasing demand for the detection of occupational hazard factors in workplaces, the national standard determination method for ammonia (sampling with absorbing solution‐analysis with Nessler reagent spectrophotometry) in the air of workplace presents many drawbacks during application in China. This review summarized the improvement and the alternate methods of the current sampling and analysis procedures for ammonia, aiming to provide reference to establish an appropriate method for the determination of ammonia in workplace air.

**Methods:**

Scientific publications in English and Chinese and the standard methods of the Deutsche Forschungsgemeinschaft (DFG) in Germany, the National Institute for Occupational Safety and Health (NIOSH) and Occupational Safety and Health Administration (OSHA) in the United States, and Ministry of Health in China for airborne ammonia collection and analysis in the workplace were reviewed.

**Results:**

The measures to improve the current sampling and analysis procedures for ammonia in China were firstly summarized. For sampling, the decrease of absorbing solution concentration and the methanesulfonic acid solution as the alternate sampling solution were suggested. For analysis, the anti‐interference measures and the optimum reaction condition between ammonia and Nessler reagent were discussed. The alternate methods including sampling conducted using solid sorbent tubes and analysis performed by ion chromatography were then considered for the determination of ammonia.

**Conclusions:**

The methods—sampling with acid‐treated solid sorbent tubes and analysis with ion chromatography—were more suitable for the determination of ammonia in workplace air. However, some details about ammonia sampling and analysis still need further investigation.

## 1 INTRODUCTION

Ammonia, with density of 0.771 g/L under standard conditions, is a colorless gas with strong irritating odor. It is usually used as refrigerant due to low boiling point. Ammonia is also an important chemical raw material and widely used in industrial production of nitric acid, fertilizer, resin, plastics, synthetic fibers, etc.^1^, ^2^ Its widespread usage makes it a common occupational hazard factor in the air of workplace and creates a large potential for worker occupational exposure. Personal exposure to ammonia at a certain concentration could cause a strong stimulating and corrosive effect on human eyes, nose, throat, and skin.^3^, ^4^ Exposure to ammonia at the concentration of about 20‐95 mg/m^3^ in the urea fertilizer factory could induce acute respiratory symptoms and acute decline in lung function.^2^ Chronic ammonia inhalation could also cause pulmonary fibrosis and interstitial lung disease.^5^

The occupational exposure limits (OELs) of ammonia are 20 mg/m^3^ (permissible concentration‐time weighted average, PC‐TWA) and 30 mg/m^3^ (permissible concentration‐short term exposure limit, PC‐STEL) in the occupational health standard in China (GBZ 2.1‐2007). The maximum workplace concentration (MAK) of ammonia established on the condition that the working week exceeds 40 hours is 14 mg/m^3^ in Germany.^6^ In the United States, the occupational exposure limits are 19 mg/m^3^ (TWA) and 26.6 mg/m^3^ (STEL).^1^, ^7^ The common standard procedure for monitoring ammonia is performed by spectrophotometry with sampling using sulfuric acid (H_2_SO_4_) solution at different concentration [0.0025 mol/L (HJ 534‐2009^8^), 0.005 mol/L (HJ 533‐2009,^9^ GB/T18204.2‐2014^10^), and 0.5 mol/L (GBZ/T 160.29‐2004^11^)], in the field of environmental protection and occupational health in China. Also, procedures including ion‐selective electrode with sampling using 0.05 mol/L H_2_SO_4_ (GB/T 14669‐1993^12^) and ion chromatography with sampling using 0.01 mol/L hydrochloric acid (YC/T 377‐2010^13^) are conducted to monitoring ammonia in public places and cigarette plants of China, respectively.

In China, the determination of ammonia in the air of workplace always uses Nessler reagent spectrophotometry with sampling in H_2_SO_4_ solution in national standard method from GB/T 16031‐1995 in 1996 to GBZ/T 160.29‐2004 in 2004 (as shown in Table 1). Nessler reagent is an alkaline solution of potassium tetraiodomercurate (K_2_HgI_4_) prepared by potassium iodide, mercuric chloride, and sodium/potassium hydroxide. The final pH of Nessler reagent is 12.4 ± 0.1 at 25°C.^14^, ^15^ Under alkaline conditions, the iodide and mercury ions in Nessler reagent can react with ammonia to form deeper yellow complex, which has a maximum absorption at the wavelength of 420 nm.^11^ The absorbance is proportional to the content of ammonia‐nitrogen in the reaction system. The concentration of ammonia can thus be determined according to the absorbance. The optimal reaction condition between ammonia and Nessler reagent is at 20‐25°C, pH 11.8‐12.4, for 10‐30 minutes.^16^, ^17^

**Table 1:** Determination methods for ammonia of DFG in Germany, NIOSH and OSHA in the United States, and Ministry of Health in China

Publisher	Year	Method number	Sampling	Analysis	References
DFG	1991	Method no. 1, vol. 2	H_2_SO_4_ (0.005 mol/L)	spectrophotometry	12
	2005	Method no. 2, vol. 9	Acid‐treated activated carbon tubes	Ion chromatography	13
NIOSH	1977	77‐157‐A	Acid‐treated silica gel tubes	Nessler reagent spectrophotometry	14
	1994	6015 Issue 2		Indophenol blue spectrophotometry	15
	1996	6016 Issue 1		Ion chromatography	16
	2016	6016 Issue 2		Ion chromatography	3
OHSA	1977	VI‐1	H_2_SO_4_ (0.05 mol/L)	Nessler reagent spectrophotometry	14
	1985	ID‐164	H_2_SO_4_ (0.05 mol/L)	Ion selective electrode method	
	1986	ID‐188	Acid‐treated activated carbon tubes	Ion chromatography	
Ministry of Health, China	1996	GB/T 16031‐1995	H_2_SO_4_ (0.01 mol/L)	Nessler reagent spectrophotometry	17
	2004	GBZ/T 160.29‐2004	H_2_SO_4_ (0.5 mol/L)	Nessler reagent spectrophotometry	8

Although sampling in an absorbing solution and analysis by spectrophotometry are convenient without desorption step, background interference,^18^ and expensive large‐scale instruments, this method reveals many drawbacks during application process. For example, (a) samples are not suitable for long‐distance shipment due to the usage of the glass bubblers containing absorbing solution^19^, ^20^; (b) This sampling method could only be used for stationary sampling but not suitable for personal sampling; (c) Samples must be analyzed on the day of sampling; (d) The highly toxic mercuric chloride reagent was involved during analysis^11^; (e) The color reaction has significant interferences.^19^ The Deutsche Forschungsgemeinschaft (DFG) in Germany and the National Institute for Occupational Safety and Health (NIOSH) and Occupational Safety and Health Administration (OSHA) in the United States have also constantly improved the method for determining ammonia in workplace air, from spectrophotometry with absorbing solution collection to ion chromatography with solid sorbent tubes collection. The renewal processes of the DFG, NIOSH, and OSHA methods and national standard method in China for ammonia measurement are listed in Table 1. The standard method for monitoring ammonia in workplace air of China is urgently to be updated. Accordingly, the improvement in the current method and the research on the alternate method were reviewed in China.

## 2 SAMPLING

The present standard method for sample collection of airborne ammonia in workplace involved the usage of two midget fritted glass bubblers in series, each containing 5 mL H_2_SO_4_ solution with a concentration of 0.5 mol/L as absorbing solution (GBZ/T 160.29‐2004).^11^ Although samples collected by this method can be directly determined without pretreatment, the standard method for ammonia sampling in workplace reveals many drawbacks during application. For example, (a) due to the use of corrosive acid solution as absorbing solution for airborne ammonia, samples collectors need to strengthen personal protection; (b) as the sample solution may leak and the glass bubblers are fragile, samples are thus not suitable for long‐distance shipment; (c) samples must be analyzed on the day of sampling, even if stored at low temperate (0°C)^21^; (d) it is not suitable for personal sampling; (e) also, high concentration of H_2_SO_4_ solution (0.5 mol/L) may affect the following color reaction during determination. Owing to these defects in the present standard method for ammonia sampling in workplace as mentioned above in China, many measures have been investigated to improve the method for ammonia sampling.

### 2.1 Absorbing solution

Absorbing solution (0.5 mol/L H_2_SO_4_) is prepared by adding 26.6 mL H_2_SO_4_ with density of 1.84 g/mL into 1000 mL distilled water in the standard method (GBZ/T 160.29‐2004).^11^ From Table 1, the concentration of absorbing solution (H_2_SO_4_ solution) in national standard method changed from 0.01 mol/L in GB/T 16031‐1995 to 0.5 mol/L in GBZ/T 160.29‐2004.^11^, ^22^ However, the high concentration of absorbing solution with too low pH value cannot provide the alkaline conditions (at pH 11.8‐12.4) required for the color reaction between ammonia and Nessler reagent during determination.^23^ This seriously affects the analysis of ammonia in the absorbing solution. When the concentration of absorbing solution decreased to 0.01 and 0.005 mol/L (H_2_SO_4_), the color reaction between ammonia and Nessler reagent is more sensitive.^24^ Because the decrease of acid concentration is helpful to increasing the pH value of the reaction system of ammonia and Nessler reagent to the optimal range (pH 11.8‐12.4). Increasing the pH value can enhance the color intensity during the color reaction between ammonia and Nessler reagent and is thus beneficial to the analysis of ammonia by spectrophotometry.

Moreover, H_2_SO_4_ solution concentration is also lower in the other standard methods [0.0025 mol/L (HJ 534‐2009)^8^ and 0.005 mol/L (HJ 533‐2009,^9^ GB/T18204.2‐2014^10^)] in China, DFG method [0.005 mol/L (Method No. 1,Vol. 2)]^25^ and OHSA method [0.005 mol/L (VI‐1 in 1977 and ID 164 in 1985)]^19^ for sampling of ammonia in the exhaust gas and air of environment, public places, and workplaces. So, the concentration of H_2_SO_4_ solution (0.5 mol/L) for sampling of airborne ammonia in workplace should be decreased to 0.01 or 0.005 mol/L.

In addition, H_2_SO_4_ is a dangerous chemical controlled by the Ministry of Public Security of China, it is inconvenient to be purchased. The alternate absorbing solution, 15 mM methanesulfonic acid (CH_4_O_2_S) solution, for ammonia sampling was considered in some researches. The sampling efficiency of CH_4_O_2_S solution in the front midget‐fritted glass bubbler could reach more than 95%, both for short and long‐term sampling of ammonia, meeting the requirement (>90%) of the standard (GBZ/T 210.4‐2008).^26^, ^27^, ^28^ It indicated that the CH_4_O_2_S solution (15 mM) could be used as the alternate sampling solution for airborne ammonia. CH_4_O_2_S is more convenient to be purchased than H_2_SO_4_ in China, because it is not a dangerous chemical controlled by the Ministry of Public Security of China. Furthermore, CH_4_O_2_S is not an oxidant, although its acid strength is similar to that of H_2_SO_4_.^29^ However, the CH_4_O_2_S solution is also a corrosive liquid.

### 2.2 Sampling with solid sorbent

#### 2.2.1 Solid sorbent tube

As a result of the inconvenience of sampling solution, the solid sorbent tube with the glass tube containing silica gel or carbon was studied for ammonia sampling. The collection efficiency of the commercial silica gel tubes (200 mg/100 mg) for ammonia could reach 100% with 0.01 mol/L H_2_SO_4_ solution as desorption solution. Meanwhile, the desorption efficiency reached 94.3%‐98.6% and the breakthrough capacity was more than 378.4 μg ammonia, permitting sampling 6 L of samples for 2 hours at the concentration of two times the threshold limit value (2 TLV, TLV in China: 20 mg/m^3^). Moreover, the samples could be stored for 14 days after collection at room temperature.^30^ The similar results were also obtained by the commercial silica gel tubes (100 mg/60 mg) with 10 mM CH_4_O_2_S solution as desorption solution.^31^ However, the breakthrough capacity of the commercial silica gel tubes was much lower than the H_2_SO_4_‐treated silica gel sampling tubes used in NIOSH method (more than 3862 μg ammonia with 63.4 L sample volume).^32^ In addition, there were other studies reported that the collection efficiency of the commercial silica gel tubes decreased clearly with increasing sampling time even at low concentration (10.6 mg/m^3^) sampling 12 L samples, with 15 mM CH_4_O_2_S solution as desorption solution determined by ion chromatography.^26^, ^27^

The carbon tubes are widely used in the collection of volatile organic carbon in workplace air. For ammonia collection in air, Asada et al (2004) reported the collection efficiency of porous carbon carbonized at 500°C with 3 mM nitric acid (HNO_3_) as desorption solution could reach 102.5% and 96.5% at 0.76 and 7.6 mg/m^3^, respectively. The recovery of activated carbon with fewer acidic functional groups was much lower than the porous carbon carbonized at 500°C, due to the lower chemical adsorption.^20^ There are few studies on the carbon tubes for the collection of ammonia in China. Ammonia sampling with tubes packed with carbon and silica gel mainly depends on the physical adsorption. As stated above, the breakthrough capacity of tubes packed with carbon and silica gel for ammonia sampling may be easily reached. It indicated that compared with chemical adsorption for ammonia, the physical adsorption capacity of tubes packed with carbon and silica gel for ammonia may be limited and breakthrough may easily occur. Therefore, the solid sorbent tubes containing silica gel and carbon are not suitable for airborne ammonia collection.

#### 2.2.2 Acid‐treated solid sorbent tube

Considering the alkaline property of ammonia, acid‐treated solid sorbent tube can be used to collect ammonia by chemical adsorption between an acid and a base. Acid‐treated silica gel and activated carbon have higher adsorption capacity for ammonia. The saturated adsorption capacity of silica gel and activated carbon impregnated with phosphoric acid for ammonia could be up to 45.2 mg/g and 34.3 mg/g, respectively.^33^ The collection efficiency of the commercial acid‐treated silica gel for ammonia could reach more than 98%, determined by ion chromatography or spectrophotometry. Desorbed with 15 mM CH_4_O_2_S solution, the desorption efficiency was 91.1%. The samples could be stored for 14 days after collection at room temperature.^26^

NIOSH manual analytical methods always use self‐made H_2_SO_4_‐treated silica gel tubes (200/100 mg) to collect ammonia (Table 1). The average recovery for ammonia in 30 L air samples reached 93.6%, 96.2%, and 103% and the desorption efficiency with deionized water as desorption solution reached 107.4%, 105.3%, and 106.9%, at the concentration of 0.5 TLV, 1 TLV, and 2 TLV (TLV: 27 mg/m^3^ for NIOSH), respectively. The breakthrough of H_2_SO_4_‐treated silica gel tubes did not occur with sampling for 317 minutes at the concentration of 2 TLV. Samples can be stored at least 7 days at room temperature and 35 days at 5°C.^7^, ^32^

OSHA method (ID‐188) uses self‐made H_2_SO_4_‐treated activated carbon tubes to substitute the inconvenient sampling solution in the former Method VI‐1 and ID‐164 for ammonia collection (as shown in Table 1). The average recovery for ammonia was up to 100% at the concentration of 2 TLV (TLV: 27 mg/m^3^ for OHSA). The breakthrough of H_2_SO_4_‐treated carbon tubes did not occur with sampling for 335 minutes. Samples can be stored at least 29 days at room temperature (20‐25°C).^19^ Similar to OSHA method, the DFG method for collecting ammonia in Germany also uses H_2_SO_4_‐impregnated activated carbon in method no. 2 with 0.0045 mol/L H_2_SO_4_ solution as desorption solution to replace H_2_SO_4_‐absorbing solution in method no. 1.^25^, ^34^ It indicated that solid sorbent tube containing acid‐treated silica gel or activated carbon can be used to collect airborne ammonia. In addition, it is remarkable that the deionized water is used as desorption solution in both NIOSH and OHSA methods. It is more safe and environmental‐friendly compared to acid solution used in other studies.

### 2.3 Comparison of absorbing solution and solid sorbent

To summarize, the respective advantages and disadvantages of absorbing solution and solid sorbent for sampling of ammonia in the air of workplace are listed in Table 2. Although samples collected by absorbing solution do not need pretreatment before analysis, there are many disadvantages for ammonia sampling with absorbing solution, as shown in Table 2. For example, (a) usage of corrosive acid solution,^11^ (b) not suitable for long‐distance shipment,^19^, ^20^ (c) samples must be analyzed on the day of sampling^11^ and (d) only be used for stationary sampling and not applicable to personal sampling. Ammonia sampling with solid sorbent tubes can overcome these defects. This method is (a) applicable to long‐distance shipment, (b) applicable to stationary sampling and personal sampling, (c) suitable for long‐time storage of samples. However, samples need desorption before analysis and desorption efficiency need be considered, when samples are collected with solid sorbent tubes.

**Table 2:** The advantages and disadvantages of absorbing solution and solid sorbent for ammonia sampling

Sampling method	Advantages	Disadvantages
Absorbing solution	Sample analysis without pretreatment.	Sample collectors need to strengthen personal protection due to the use of corrosive acid solution; Not suitable for long‐distance shipment; Samples must be analyzed on the day of sampling; Not applicable to personal sampling.
Solid sorbent	Applicable to long‐distance shipment; Applicable to personal sampling; Long‐storage time of samples.	Samples need desorption before analysis; Desorption efficiency need be considered.

## 3 ANALYSIS

In national standard method of China from GB/T 16031‐1995 in 1996 to GBZ/T 160.29‐2004 in 2004, Nessler reagent spectrophotometry is always used to detect the concentration of ammonia in the air of workplace (Table 1). However, this detection method involved the use of mercuric chloride reagent, which is highly toxic and easily causes adverse effects on tester and environment. The subsequent treatment of waste liquid is also difficult. In addition, color reaction could be easily affected by the interferent and the conditions (such as temperature, time, pH, etc). As a result of these drawbacks of the Nessler reagent spectrophotometry, the improvement to this method and the alternate method was investigated in China.

### 3.1 Improvement in the Nessler reagent spectrophotometry

To avoid the interferences (such as Fe^3+^ and sulfate), the sodium potassium tartrate solution was added into the sample solution before the addition of Nessler reagent to remove these interferents and make the absorbance value more stable.^35^ The addition of dilute hydrochloric acid (HCl) before colorimetric reaction could be used to eliminate the interference of formaldehyde.^24^ The interference of hydrogen sulfide can be eliminated by adding lead acetate cotton before the sampling tube.^11^ In addition, color reaction is affected by the reaction condition, such as temperature and time. When the temperature was 5‐15°C, it was too low to ensure the complete color reaction. The temperature at 30°C was too high resulting in fading. The optimum temperature was 20‐25°C to ensure the complete reaction and reliable results. Similarly, the coloration time could influence the color reaction between Nessler reagent and ammonia. If the time was less than 10 minutes, it was too short leading to incomplete reaction. If the time was more than 30 minutes, the color deepened first and then faded. The optimum time was 10‐30 minutes to ensure the stable result.^24^

### 3.2 Ion chromatography

Ion chromatography is the alternate method for the determination of ammonia, due to its advantages comparing with spectrophotometry, such as high sensitivity, good selectivity, avoiding the use of highly toxic chemical reagents, and easy to realize automation.^1^

#### 3.2.1 Conditions of ion chromatography

Ion chromatography for ammonia measurement was performed with ion chromatograph equipped with cation separator column, cation guard column, cation suppressor column, and conductivity detector. For eluant, CH_4_O_2_S, H_2_SO_4_, and HCl solutions were usually considered in China. The flow rate of eluant was usually set to 1 mL/min. Compared with CH_4_O_2_S and H_2_SO_4_ solution, HCl solution (20 mM) could affect the retention time of ammonia, since it was volatile and its concentration fluctuated greatly.^27^ CH_4_O_2_S and H_2_SO_4_ solution with concentration of 15 mM as eluant could be beneficial to the separation of the peaks of mix ions containing NH_4_^+^, Na^+^, K^+^, Mg^2+^, and Ca^2+^.^27^ However, H_2_SO_4_ as dangerous chemical is inconvenient to be purchased as stated above. CH_4_O_2_S solution was also a corrosive liquid and could not separate the interfering peaks of monomethylamine, monoethylamine, and dimethylamine.^27^ HNO_3_ solution at 3 mM was also used as eluant for the determination of ammonia.^20^ The separation effect of peaks with HNO_3_ solution as eluant should be further investigated.

In NIOSH manual analytical methods (6016 Issue 1 and 2) for the determination of ammonia by ion chromatography, 48 mM HCl/4 mM 2,3‐diaminopropionic acid monohydrochloride/4 mM L‐histidine monohydrochloride monohydrate (HCl/DAP‐HCl/L‐histidine‐HCl) is used as eluant and 12 mM HCl/0.25 mM DAP‐HCl/0.25 mM L‐histidine‐HCl as alternate eluant to avoid the interference of alkanolamine.^7^, ^36^ In OSHA method (ID‐188), HCl/DAP‐HCl/L‐histidine‐HCl solution with low and high concentration is used as weak and strong eluant, which is the same as that in NIOSH method 6016. In addition, 12 mM HCl can be used as the alternate eluant to offer sufficient resolution between ammonia and methyl‐ or dimethylamine.^19^ However, the DFG method in Germany applies H_2_SO_4_ solution as eluant.^34^

#### 3.2.2 Precision and accuracy

The precision and accuracy of ion chromatography for the determination of ammonia was considered. As ammonia was collected both with CH_4_O_2_S and H_2_SO_4_ absorbing solution and with solid sorbent tubes, the average recovery was 95%‐105% and the relative standard deviation (RSD) of intra‐day and inter‐day determination of NH_4_^+^ at different concentration (9‐100 μg/mL) was less than 10%.^26^, ^27^, ^31^, ^37^ The precision and accuracy can meet the requirements (RSD ≤10%, the average recovery: 95%‐105%) of the determination methods of chemical substances in the air of workplace.^28^ In addition, there was no significant difference between the results determined by ion chromatography and Nessler reagent spectrophotometry for NH_4_^+^ reference material or airborne ammonia collected by absorbing solution and solid sorbent tubes.^26^, ^37^ It indicated that ion chromatography can be used for the determination of ammonia in the air of workplace.

### 3.3 Comparison of the Nessler reagent spectrophotometry and ion chromatography

The advantages and disadvantages of the Nessler reagent spectrophotometry and ion chromatography for ammonia analysis are listed in Table 3. Although the cost is relatively lower, the Nessler reagent spectrophotometry for the determination of ammonia exposed many defects during application, such as (a) use of highly toxic reagent (mercuric chloride), (b) difficulty in subsequent treatment of waste liquid,^11^ (c) easily affected by reaction conditions (such as temperature, time, pH, etc),^24^ (d) easily affected by the interferent (such as formaldehyde, hydrogen sulfide and so on).^35^ The ion chromatography for ammonia analysis can overcome the shortcomings of the Nessler reagent spectrophotometry by (a) avoiding the use of highly toxic chemical reagents, (b) avoiding subsequent treatment of waste liquid, (c) being easy to realize automatic injection and time‐saving. Moreover, the ion chromatography for the determination of ammonia presents a high sensitivity and good selectivity. One disadvantage of the ion chromatography for the determination of ammonia is that monoethanolamine, isopropanolamine, or propanolamine would produce peaks in the vicinity of the ammonium ion constituting a positive interference.^19^

**Table 3:** The advantages and disadvantages of the Nessler reagent spectrophotometry and ion chromatography for ammonia analysis

Analysis method	Advantages	Disadvantages
Nessler reagent spectrophotometry	No expensive equipment required.	Use of highly toxic reagent (mercuric chloride); Difficulty in subsequent treatment of waste liquid; Easily affected by reaction conditions (such as temperature, time, pH, etc); Easily affected by the interferent (such as formaldehyde, hydrogen sulfide, and so on).
Ion chromatography	Avoiding the use of highly toxic chemical reagents; Avoiding subsequent treatment of waste liquid; Easy to realize automatic injection and time‐saving; High sensitivity and good selectivity.	May be interfered by monoethanolamine, isopropanolamine, or propanolamine.

## 4 CONCLUSIONS

With the increasing requirement of the detection of occupational hazard factors in the air of workplace, the current national standard method (0.5 mol/L H_2_SO_4_ solution collection‐Nessler reagent spectrophotometry) for monitoring ammonia presents many defects and needs to be updated in China. The measures to improve the current sampling and analysis procedures for ammonia in China were firstly summarized. For sampling, the decrease in absorbing solution (H_2_SO_4_ solution) concentration and the CH_4_O_2_S solution (15 mM) as the alternate sampling solution were suggested. For analysis, the anti‐interference measures and the optimum reaction condition (at 20‐25°C for 10‐30 minutes) between ammonia and Nessler reagent were discussed. Although the improvement in the current method was investigated, some drawbacks still exists, such as only 1 day storage time for samples, inapplicability for long‐distance shipment, and personal sampling. Then, the alternate methods for sampling and analysis were considered. The methods for ammonia collection and analysis of DFG in Germany, NIOSH and OSHA in the United States were also involved. The method containing sampling conducted using acid‐treated solid sorbent tubes and analysis performed by ion chromatography were more suitable for the determination of ammonia in workplace air.

However, some details about ammonia collection and analysis still need further investigation for establishing an appropriate method for the determination of ammonia in China, such as sampling tubes (packed with acid‐treated silica gel or activated carbon), desorption solution of samples and eluant during analysis with ion chromatography. The acid‐treated silica gel tubes used in some studies of China were not specific to ammonia collection. The background level of NH_4_^+^ and other cations fluctuated greatly, interfering with the detection results and affecting the column efficiency of ion chromatograph. The CH_4_O_2_S solution usually used as desorption solution and eluant in some studies of China is corrosive and could not eliminate the interference of amines. We can refer the methods of NIOSH and OSHA for ammonia sampling and analysis, making efficient and practical sampling tubes specific to ammonia collection and choosing more safe and environmental‐friendly desorption solution and eluant.

## DISCLOSURE

*Approval of the research protocol:* NA. *Informed Consent*: NA. *Registry and the Registration No. of the study/Trial*: NA. *Animal Studies*: NA. *Conflict of Interest*: The authors declare no conflict of interest.

## AUTHOR CONTRIBUTIONS

Zhizhen Xu conceived the ideas and drafted the text; Dongxu Wang and Zhe Bi collected the data; Ling Guo revised the text; Zhaohui Fu conceived the ideas and gave the final approval of the manuscript.
